# One-year cumulative live birth rate associated with the number of oocytes in ovarian stimulation with follitropin delta: a pooled analysis of four randomized controlled trials

**DOI:** 10.1093/humrep/deaf111

**Published:** 2025-06-12

**Authors:** Rita Lobo, Samuel Santos-Ribeiro, Ali Falahati, Kelle Moley, Anja Pinborg, Nick S Macklon, Ida E Jepsen

**Affiliations:** Ferring Pharmaceuticals, Global Research and Medical, Copenhagen, Denmark; IVI Lisbon, Department of Obstetrics and Gynaecology, Faculty of Medicine, University of Lisbon, Lisbon, Portugal; Ferring Pharmaceuticals, Global Biometrics, Copenhagen, Denmark; Ferring Pharmaceuticals, Global Research and Medical, Copenhagen, Denmark; Fertility Clinic, Department of Gynaecology, Fertility and Obstetrics, Rigshospitalet, Copenhagen University Hospital, Copenhagen, Denmark; London Women’s Clinic, London, UK; Ferring Pharmaceuticals, Global Research and Medical, Copenhagen, Denmark

**Keywords:** cumulative live birth rate, oocytes number, follitropin delta, ovarian stimulation, fresh and frozen cycles

## Abstract

**STUDY QUESTION:**

What number of oocytes retrieved is associated with the highest cumulative live birth rates (CLBRs) in the fresh and subsequent frozen cycles following ovarian stimulation with follitropin delta?

**SUMMARY ANSWER:**

The CLBR increased with the number of oocytes retrieved, plateauing at 21–25 oocytes.

**WHAT IS KNOWN ALREADY:**

Live birth rate (LBR) per fresh cycle is the conventionally reported outcome of IVF; however, the marked increase in cryopreserved cycles in recent years suggests that the CLBR has emerged as a more relevant outcome. In the fresh cycle, the number of oocytes retrieved is regarded as a prognostic factor for LBR, and a similar association has been shown for CLBR.

**STUDY DESIGN, SIZE, DURATION:**

Pooled analysis including 1746 patients from four randomized controlled trials. Trials were identified from clinical trials available in the Ferring Pharmaceuticals database up to June 2023. Selected trials used follitropin delta for ovarian stimulation and collected outcome data from both fresh and frozen cycles. Follitropin delta dose–response trials, as well as trials investigating follitropin delta in repeated ovarian stimulation cycles, were excluded. Patients included in the analysis underwent ovarian stimulation with follitropin delta and had at least one oocyte retrieved. The outcome of CLBR in the fresh and subsequent frozen cycles was evaluated in relation to the number of oocytes retrieved. CLBR was calculated as the number of patients with at least one live birth divided by the number of all patients included in the analysis.

**PARTICIPANTS/MATERIALS, SETTING, METHODS:**

Trial participants were women, 18–42 years of age, who were undergoing their first or second IVF/ICSI cycle in a GnRH antagonist/agonist protocol. Triggering was performed with hCG or GnRH agonist, and insemination was performed by IVF or ICSI. Single or double blastocyst transfer was performed on Day 5 in the fresh cycle, and all viable surplus blastocysts were cryopreserved on Day 5 or Day 6. All pregnancies from the fresh cycle and frozen cycles initiated within 1 year after the start of stimulation were followed until birth. The association between the number of oocytes retrieved and CLBR was assessed using a logistic regression analysis with fractional polynomials to obtain predicted CLBR. Subgroup analyses were performed based on age, anti-Müllerian hormone (AMH), and number of oocytes retrieved.

**MAIN RESULTS AND THE ROLE OF CHANCE:**

Overall, 15 trials with follitropin delta were identified in the database. Of those, 11 trials were not eligible, and the remaining 4 trials were included. In total, 1746 patients were included in the analysis. The mean age was 33.8 years (range 21–42 years), and the median AMH level was 17.0 pmol/l (range 0.3–164.2 pmol/l). The vast majority of patients (1645 patients, 94.2%) were treated with a GnRH antagonist protocol, while 101 patients (5.8%) were treated with a GnRH agonist protocol. Overall, 1541 patients (88.3%) received hCG triggering, and 205 patients (11.7%) received GnRH agonist triggering. Frozen cycles (maximum of six) were initiated by 740 patients (42.4%). The study population underwent a total of 2948 cycles: 1746 fresh cycles (referring to ovarian stimulation cycles, with or without transfer) and 1202 frozen cycles (initiated cycles, with or without transfer). The mean number of oocytes retrieved was 12.4 (range 1–72), the fresh cycle LBR was 29.1%, and the CLBR was 51.4%. The CLBR increased with the number of oocytes retrieved up to a plateau starting at 21–25 oocytes. The CLBR reached above 60% at >15 oocytes and above 70% at >20 oocytes. The CLBR decreased with increasing age (57.1%, 51.6%, and 35.8% at <35, 35–37, and ≥38 years), while it was similar for AMH <15 and ≥15 pmol/l (52.0% and 50.9%, respectively). A continued increase in predicted CLBR from 15 oocytes retrieved was observed in older patients (≥38 years); from 41.3% to 53.4% to 58.7% at 15–19, 20–24, and ≥25 oocytes. No equivalent benefit was observed in younger patients (<38 years), where corresponding rates were 72.5%, 68.0%, and 78.8% in patients <35 years and 70.3%, 73.1%, and 71.5% in patients 35–37 years. The fresh cycle LBR decreased beyond 14 oocytes, while the CLBR continued to increase by the number of oocytes retrieved.

**LIMITATIONS, REASONS FOR CAUTION:**

A limited number of patients included in the analysis had ≥20 oocytes retrieved (249 patients, 14.3%).

**WIDER IMPLICATIONS OF THE FINDINGS:**

This analysis suggests an increase in CLBR with the number of oocytes retrieved up to a plateau starting at 21–25 oocytes following ovarian stimulation cycles with follitropin delta and subsequent frozen cycles. An increase in CLBR from 20 oocytes was evident in older but not in younger patients.

**STUDY FUNDING/COMPETING INTEREST(S):**

The study was funded by Ferring Pharmaceuticals A/S, Copenhagen, Denmark. S.S.-R. has received research funding from Organon/MSD, Theramex, and Gedeon-Richter, consulting fees from Organon/MSD, Ferring Pharmaceuticals, Merck Serono, and IBSA, payment or honoraria from Organon/MSD, Besins and Gedeon-Richter, support for attending meetings from Gedeon-Richter and Besins, is an advisory board member for TTRANSPORT and Deputy of the ESHRE SQART SIG, and owns stocks of IVI Lisboa, Clinica de Reprodução assistida Lda. A.P. has received grants from Cryos, grants and payments from Gedeon Richter, Ferring Pharmaceuticals and Merck A/S, payments from Organon, consulting fees from IBSA, Ferring Pharmaceuticals, Gedeon Richter, Cryos, and Merck A/S, and travel support from Gedeon Richter. N.S.M. has received speaker and consultancy fees from Ferring Pharmaceuticals, IBSA, Merck, Freya, and Gedeon Richter, and is a shareholder in Verso Biosense. K.M. has been a scientific advisor for Calla Lily Clinical Care, Evvy, and AutoIVF. R.L., A.F., K.M., and I.E.J. are employees of Ferring Pharmaceuticals.

**REGISTRATION NUMBER:**

N/A.

## Introduction

The number of oocytes retrieved is regarded as a predictor of live birth rates (LBRs) following fresh embryo transfer in ovarian stimulation for IVF. Studies have shown that LBRs either plateau or decline after a particular number of oocytes are retrieved, and optimum yields in the range between 6 and 15 oocytes have been suggested, taking the risk of ovarian hyperstimulation syndrome (OHSS) in high responders into consideration (van der Gaast *et al.*, 2006; Sunkara *et al.*, 2011; Ji *et al.*, 2013; Steward *et al.*, 2014; Magnusson *et al.*, 2018; Polyzos *et al.*, 2018; Lobo *et al.*, 2025).

The LBR per fresh cycle is the conventionally reported outcome of IVF. In recent years, there has been a marked increase in cryopreservation of embryos and subsequent frozen embryo transfers (FETs). The switch of cryopreservation method from slow freeze to vitrification, as well as embryo culture to the blastocyst stage, has led to a significant increase in embryo cryo-survival rates (Saket *et al.*, 2021). Another factor behind the rise in FET cycles is the increase in single embryo transfers (to avoid the potential complications of multiple pregnancies), resulting in more excess embryos available for cryopreservation. In addition, ‘freeze-all’ cycles have emerged as an alternative to fresh embryo transfer cycles, initially as a strategy in combination with GnRH agonist triggering to avoid OHSS in high responders (Devroey *et al.*, 2011), but also used with preimplantation genetic testing, or to overcome asynchrony of embryo and endometrial receptivity (Shapiro *et al.*, 2008). The increase in number and successful outcomes of FET cycles suggests that the cumulative live birth rate (CLBR) has arisen as a more clinically relevant outcome than fresh cycle LBR in IVF treatment.

Studies imply that CLBR continues to increase with number of oocytes retrieved beyond the plateau or decline observed for fresh cycle LBRs (Ji *et al.*, 2013; Drakopoulos *et al.*, 2016; Toftager *et al.*, 2017; Magnusson *et al.*, 2018; Polyzos *et al.*, 2018; Law *et al.*, 2019; Neves *et al.*, 2023).

This study aimed to investigate the association between the number of oocytes retrieved and CLBR in a pooled analysis of four randomized controlled trials using the recombinant FSH follitropin delta for ovarian stimulation. The trials included one stimulation cycle and assessed live birth from the fresh cycle and subsequent cryopreserved cycles initiated within 1 year of the ovarian stimulation.

## Materials and methods

**Figure 1.**
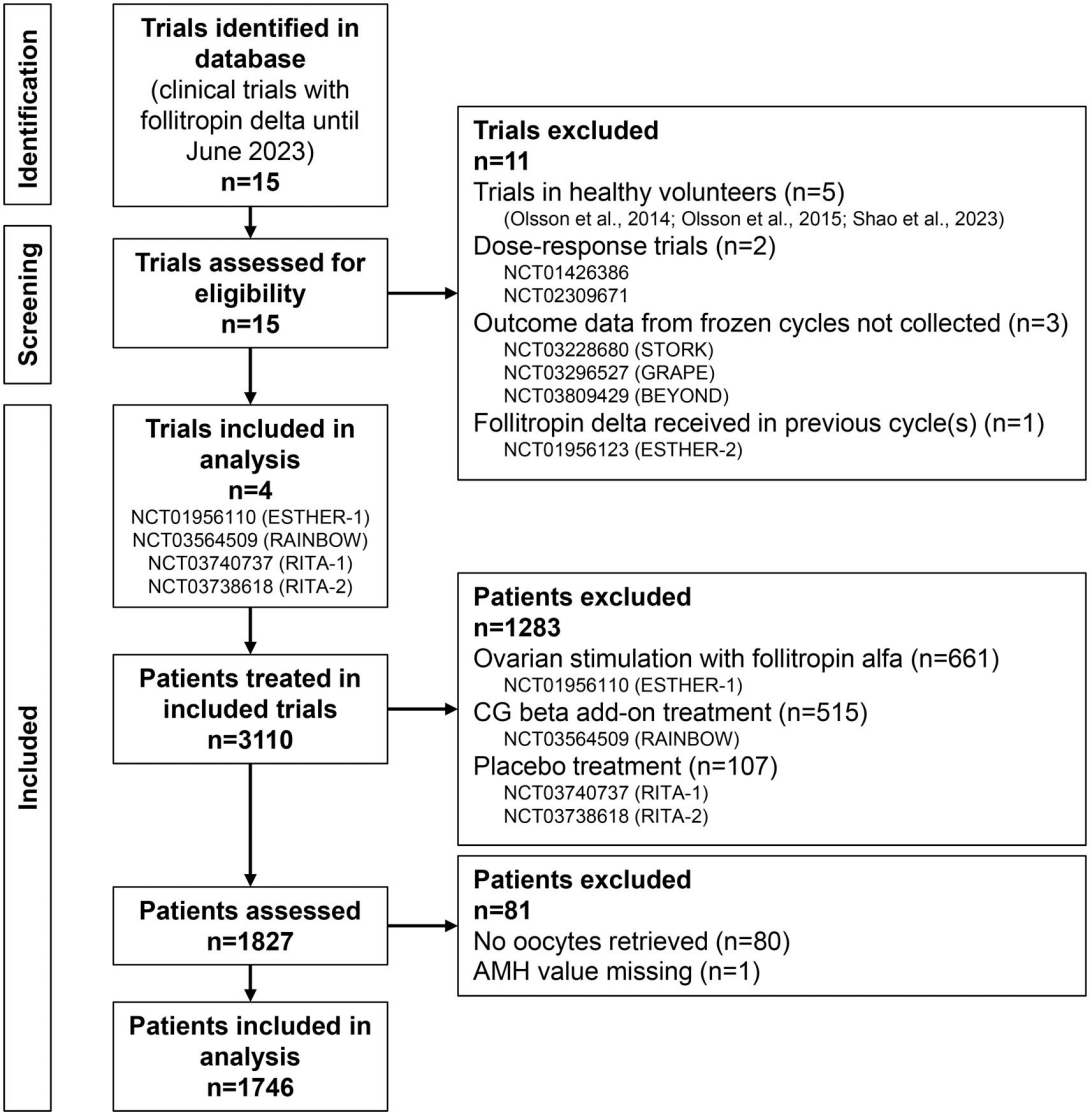
**Selection of trials and patients.** AMH, anti-Müllerian hormone; CG, chorionic gonadotropin. Olsson *et al*., 2014, 2015; Shao *et al*., 2023.

This study is a pooled analysis investigating the association between number of oocytes retrieved and CLBR in 1746 patients from four randomized controlled trials (trial registration numbers NCT01956110 [ESTHER-1], NCT03564509 [RAINBOW], NCT03740737 [RITA-1], and NCT03738618 [RITA-2]) conducted in 12 countries in Europe, North America, and South America. The trials were identified (without a registered protocol) from clinical trials available in the Ferring Pharmaceuticals database up to June 2023 and were selected based on the use of follitropin delta for ovarian stimulation and the collection of outcome data from both fresh and frozen cycles as part of the study design. Follitropin delta dose–response trials were excluded as well as trials investigating follitropin delta in repeated ovarian stimulation cycles (Fig. 1).

The original trial protocols and results have been described previously (Nyboe Andersen *et al.*, 2017; ClinicalTrials.gov, Identifier NCT03740737, 2018; ClinicalTrials.gov, Identifier NCT03738618, 2018; Fernandez Sanchez *et al.*, 2022; Doody *et al.*, 2023; Scheiber *et al.*, 2023; Grover *et al.*, 2024). The trials had obtained regulatory and ethical approval and were performed in accordance with the principles of the Declaration of Helsinki, the International Conference on Harmonisation Guidelines for Good Clinical Practice, and local regulatory requirements. Written informed consent was obtained from all participants.

### Study participants

The trials included women undergoing their first or second IVF/ICSI cycle (no women had previously received follitropin delta). The women were diagnosed with unexplained infertility, tubal infertility, endometriosis stage I/II, or had partners with male factor infertility. They were 18–42 years of age, had a BMI of 17.5–38.0 kg/m^2^, and regular menstrual cycles of 24–35 days. The exclusion criteria included endometriosis stage III–IV and a history of recurrent miscarriage. Patients included in the current analysis were treated with follitropin delta and had at least one oocyte retrieved. Overall, 1827 patients received follitropin delta in the four clinical trials. One patient with a missing anti-Müllerian hormone (AMH) value and 80 patients with no oocytes retrieved were excluded from the analysis. The remaining 1746 patients had at least one oocyte retrieved and were included in the analysis. Because of differences in the choice of comparators between the trials, comparator data were not included in the analysis (Fig. 1).

### Study procedures

Ovarian stimulation was performed with follitropin delta (REKOVELLE^®^, Ferring Pharmaceuticals). The patients either received an individualized dosing of follitropin delta at a fixed daily subcutaneous dose determined by their serum AMH concentration at screening and body weight at randomization/stimulation Day 1 (NCT01956110 and NCT03564509; the individualized dosing algorithm of follitropin delta is detailed in Nyboe Andersen *et al.*, 2017), or they followed a dosing regimen with a daily starting dose of 12 or 15 µg, which could be adjusted in increments of 3 µg after the first 4 stimulation days (NCT03740737 and NCT03738618).

Patients either followed a GnRH antagonist protocol, with follitropin delta initiated on Day 2–3 of the menstrual cycle and GnRH antagonist (0.25 mg daily) initiated on stimulation Day 5 or 6 and continued throughout stimulation, or a GnRH agonist protocol, with GnRH agonist (0.1 mg daily) started in the mid-luteal phase of the menstrual cycle and follitropin delta initiated after 14 days.

Triggering of final follicular maturation was performed when ≥3 follicles or ≥2 follicles with a diameter ≥17 mm were observed. Triggering was performed with hCG if <25 follicles or <20 follicles ≥12 mm were observed. If ≥25 follicles or ≥20 follicles ≥12 mm were observed, triggering was performed with a GnRH agonist (not applicable for the GnRH agonist protocol), or the cycle was cancelled (when >25 follicles or >35 follicles ≥12 mm were observed, depending on the trial). If the investigator judged that the triggering criterion could not be reached by Day 20, the cycle was cancelled (in one of the trials, triggering could still be performed if at least 1 follicle ≥17 mm was observed).

Oocyte retrieval took place 36 ± 2 h after triggering. Insemination was performed by IVF or ICSI, and single or double blastocyst transfer was performed on Day 5. Single blastocyst transfer was mandatory for patients ≤37 years or ≤34 years (depending on the trial), whereas in patients ≥38 years or ≥35 years (depending on the trial), single blastocyst transfer was performed if a good-quality blastocyst was available; otherwise, double blastocyst transfer was performed. All viable surplus blastocysts were cryopreserved. For patients who were triggered with a GnRH agonist, all blastocysts were cryopreserved, and no transfer was performed in the fresh cycle. All pregnancies from the fresh cycle and frozen cycles initiated within 1 year after the start of stimulation were followed until birth. In two of the trials, frozen cycles were performed in accordance with local clinical practice. In the other two trials, the protocol mandated either natural cycles or programmed cycles with oestradiol and progesterone. In the cryopreserved cycles, there was no defined transfer policy, and the choice between single and double blastocyst transfer was at the discretion of the investigator.

### Study outcomes

The primary outcome was cumulative live birth, defined as the birth of at least one live neonate (irrespective of gestational age) in the fresh or a subsequent frozen cycle using blastocysts derived from a single oocyte retrieval. Each patient underwent one stimulation cycle. CLBR was calculated as the number of patients with at least one live birth (only the first delivery was considered), divided by the number of all patients included in the analysis (i.e. patients treated with follitropin delta who had at least one oocyte retrieved).

The secondary outcome was live birth, defined as the birth of at least one live neonate, in the fresh cycle only. LBR was calculated as the number of patients with at least one live birth divided by the number of all patients included in the analysis.

### Statistical analysis

Continuous variables are presented as mean ± SD or median and interquartile range. Categorical variables are presented as number and percentages.

The association between the number of oocytes retrieved and cumulative live births was assessed using a logistic regression analysis with fractional polynomials to obtain predicted cumulative live births where the final model was selected using a backward selection method. The model was unadjusted. A sensitivity analysis was performed to evaluate the effect of age and AMH on the model. Furthermore, a logistic regression analysis was used to describe CLBR on one hand and the number of oocytes retrieved (grouped by 1–7, 8–14, 15–19, 20–24, and ≥25 oocytes), age (grouped by <35, 35–37, and 38–42 years), or AMH level (<15 or ≥15 pmol/l) on the other hand. Finally, subgroup analyses were performed based on follitropin delta dosing strategy (individualized dosing based on AMH level and body weight, fixed throughout stimulation [trials NCT01956110 and NCT03564509], or starting doses of 12 or 15 µg with potential adjustments during stimulation [trials NCT03740737 and NCT03738618]).

## Results

### Selection of trials

Overall, 15 trials with follitropin delta were identified in the database. Of those, five were performed in healthy volunteers (with no pregnancy outcome data), two were dose–response trials, three did not collect outcome data from frozen cycles, and one evaluated follitropin delta in repeated cycles. As a result, four trials were selected for the analysis (Fig. 1).

### Characteristics and study outcomes of the overall study population

In total, 1746 patients were included in the analysis, selected based on ovarian stimulation with follitropin delta and retrieval of at least one oocyte (Fig. 1). The mean age of the study population was 33.8 years, and the median AMH level was 17.0 pmol/l (Table 1). The most common primary reason for infertility was unexplained infertility, followed by male factor and tubal infertility, and the vast majority of patients (94.2%) were treated with a GnRH antagonist protocol. For 1575 patients (90.2%), the trial stimulation cycle was their first IVF/ICSI cycle, while for 171 patients (9.8%), it was their second IVF/ICSI cycle. Overall, 1541 patients (88.3%) received hCG triggering, and 205 patients (11.7%) received GnRH agonist triggering.

**Table 1.** Characteristics of the study population.

Characteristic	N = 1746
Age (years), mean ± SD (range)	33.8 ± 4.1 (21–42)
Age, grouped (years), n (%)	
<35	943 (54.0)
35–37	458 (26.2)
≥38	345 (19.8)
AMH (pmol/l), median (range)	17.0 (0.3–164.2)
AMH, grouped (pmol/l), n (%)	
<15	764 (43.8)
≥15	982 (56.2)
BMI (kg/m^2^), mean ± SD (range)	25.4 ± 4.7 (17.5–38.5)
Primary reason for infertility, n (%)	
Unexplained infertility	789 (45.2)
Male factor	599 (34.3)
Tubal infertility	288 (16.5)
Endometriosis stage I/II	63 (3.6)
Other	5 (0.3)
Missing	2 (0.1)
Protocol, n (%)	
GnRH antagonist	1645 (94.2)
GnRH agonist	101 (5.8)

AMH, anti-Müllerian hormone.

Of the 1746 patients, 740 patients (42.4%) initiated frozen cycles. In total, the study population underwent 2948 cycles: 1746 fresh cycles (referring to ovarian stimulation cycles, with or without transfer) and 1202 frozen cycles (initiated cycles, with or without transfer). The maximum number of frozen cycles for a patient was six (the numbers of patients initiating cryopreserved cycle 1–6 are found in Table 2). At the cut-off for data collection (1 year after start of stimulation), 279 patients (16.0% of the study population) had not achieved a live birth but had remaining cryopreserved blastocysts.

**Table 2.** Treatment outcomes of the study population.

Outcome variable	N=1746
Oocytes retrieved, mean ± SD	12.4 ± 8.4
Oocytes retrieved, grouped, n (%)	
1–7	528 (30.2)
8–14	691 (39.6)
15–19	278 (15.9)
20–24	130 (7.4)
≥25	119 (6.8)
Fresh cycle live birth, n (%)	508 (29.1)
Live birth, n/N (%)	
Cryopreserved cycle 1	262/738 (35.5)
Cryopreserved cycle 2	90/290 (31.0)
Cryopreserved cycle 3	27/114 (23.7)
Cryopreserved cycle 4	11/43 (25.6)
Cryopreserved cycle 5	0/14 (0)
Cryopreserved cycle 6	0/3 (0)
Cumulative live birth, n (%)	897 (51.4)

**Figure 2.**
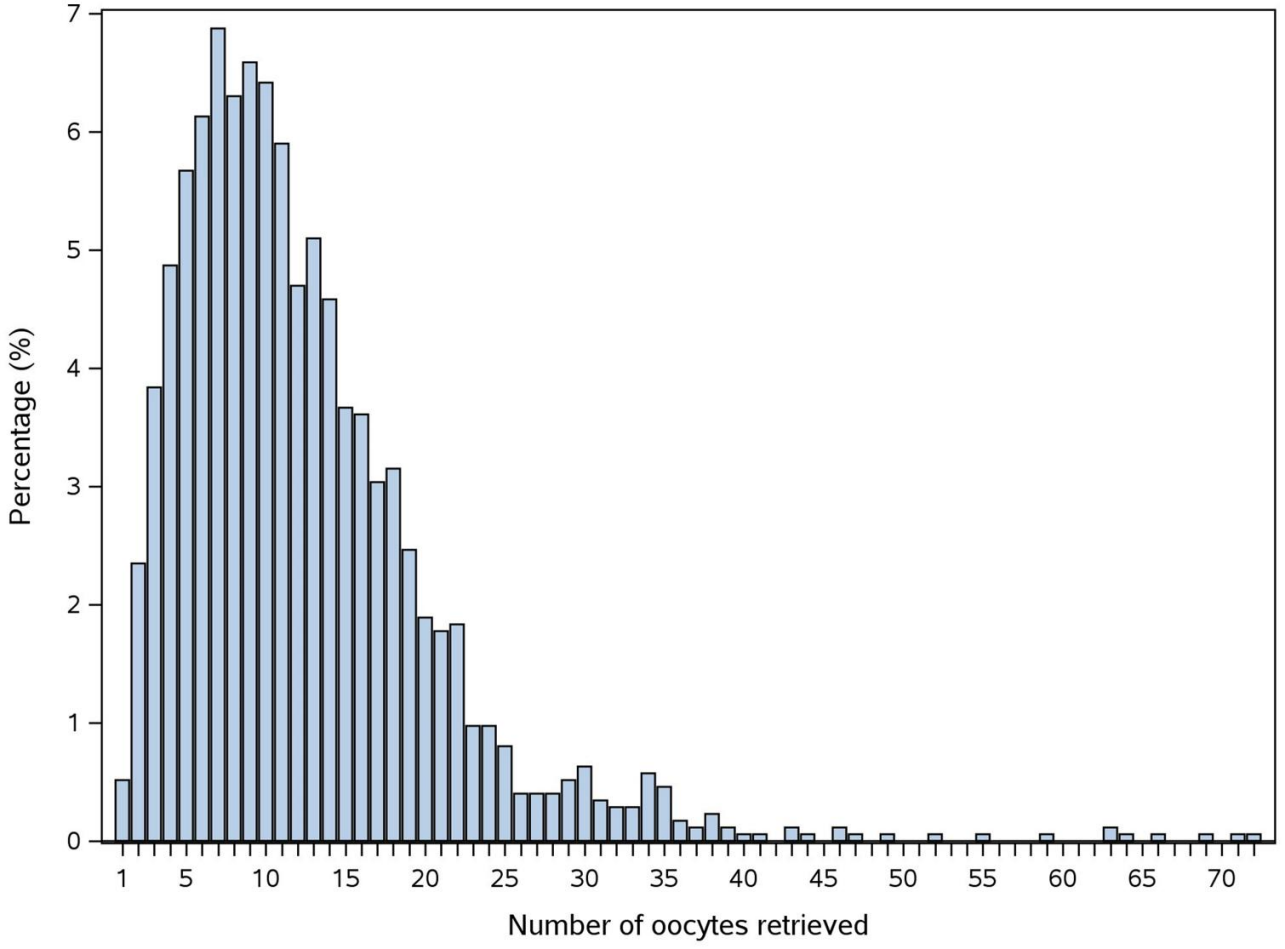
Distribution in the number of oocytes retrieved in the overall study population (N = 1746).

The study outcomes of the overall study population are presented in Table 2. The mean number of oocytes retrieved was 12.4; the distribution is illustrated in Fig. 2. Transfer in the fresh cycle was performed for 1303 patients (74.6%). Overall, 1440 patients (82.5%) had single blastocyst transfer only, 60 patients (3.4%) had double blastocyst transfer only, 85 patients (4.9%) had both single and double blastocyst transfer (in separate cycles), and 161 patients (9.2%) had no transfer. The LBR in the fresh cycle was 29.1%. The LBR decreased by the number of repeated frozen cycles, and no live birth was achieved in the fifth or sixth frozen cycle. The overall CLBR was 51.4%.

### Association between number of oocytes retrieved and cumulative live birth

**Figure 3.**
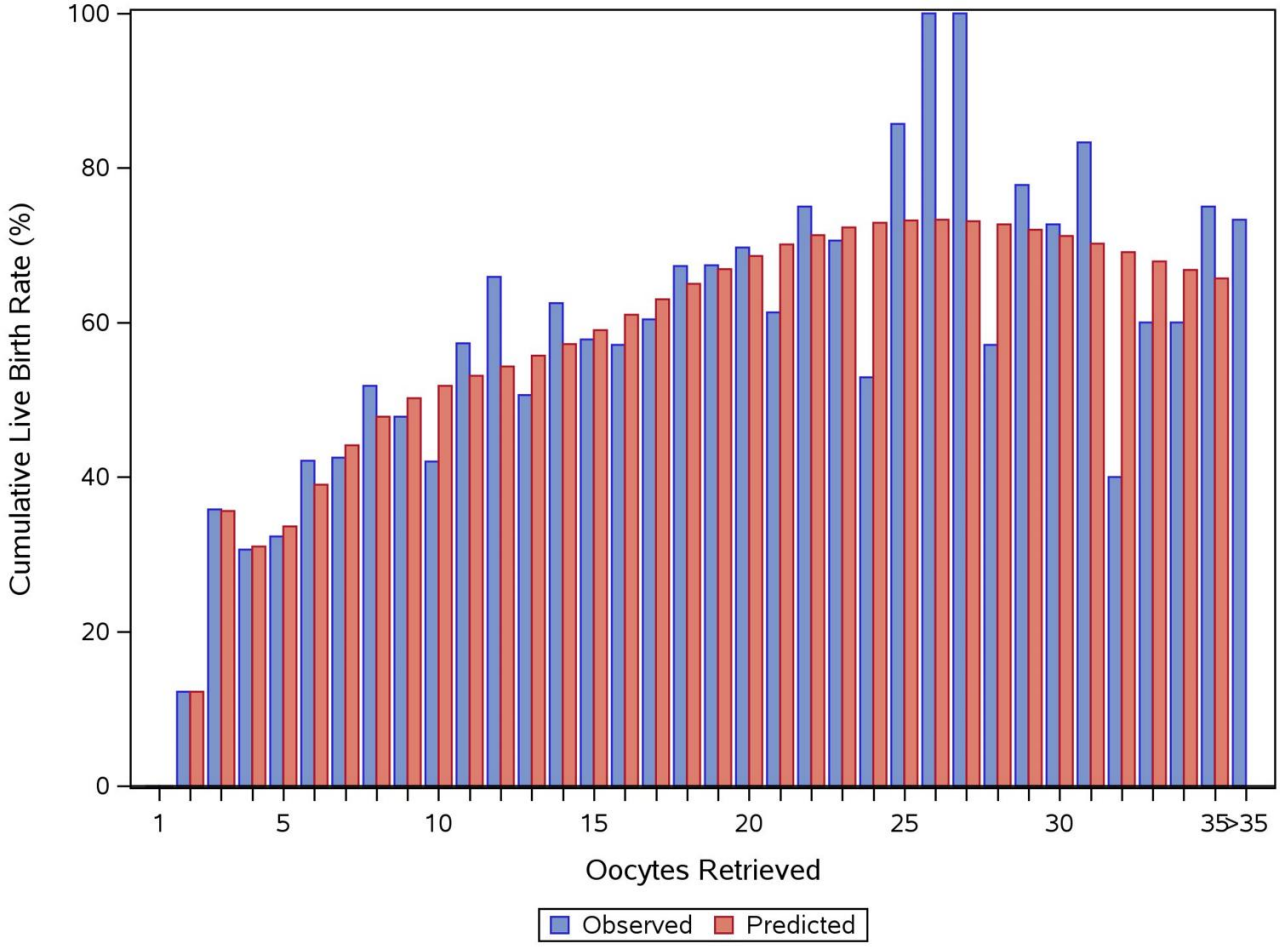
**Observed and predicted cumulative live birth rate by number of oocytes retrieved.** Blue bars represent observed rates, and red bars represent predicted rates. Predicted cumulative live birth rates were obtained using a logistic regression analysis with fractional polynomials.

Figure 3 shows the observed and predicted CLBR by number of oocytes retrieved. Due to the high distribution in the number of oocytes retrieved and the low number of patients at each fraction of the highest ovarian responses observed, the figure only displays data up to 35 oocytes. The predicted CLBR increased with the number of oocytes retrieved, reaching above 60% at >15 oocytes retrieved and above 70% at >20 oocytes retrieved. At 21–25 oocytes, the CLBR started to plateau. Sensitivity analysis of the model, including adjustments for age and AMH, did not change the conclusion of the analysis. Subgroup analyses based on dosing strategy (Supplementary Fig. S1) displayed similar trends; however, the CLBR was higher in patients treated with individualized follitropin delta compared to patients who received starting doses of 12 or 15 µg (peaks were observed at 80.7% and 72.3%, respectively), and the plateau was shifted towards higher oocyte numbers, starting at around 25 oocytes.

**Figure 4.**
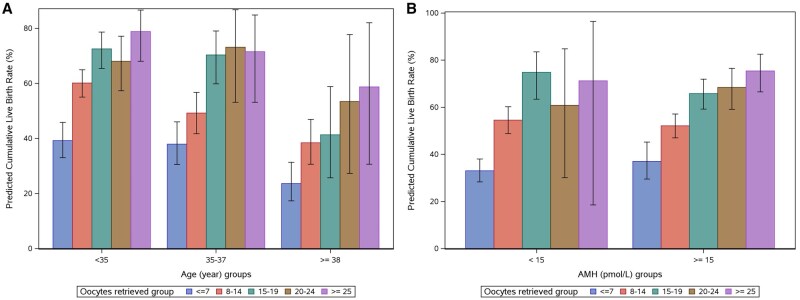
**Subgroup analyses of the cumulative live birth rate.** (**A**) Cumulative live birth rate by number of oocytes retrieved (grouped) and age group. (**B**) Cumulative live birth rate by number of oocytes retrieved (grouped) and anti-Müllerian hormone (AMH) level. Number of oocytes retrieved are grouped as ≤7 oocytes (blue bars), 8–14 oocytes (red bars), 15–19 oocytes (green bars), 20–24 oocytes (brown bars), and ≥25 oocytes (purple bars). Data are presented as percentage (95% CI). Predicted cumulative live birth rates were obtained using logistic regression analyses.

The number of patients by subgroup (based on age, AMH, and number of oocytes retrieved) is presented in Table 3. The subgroup analyses demonstrated that the CLBR decreased with increasing age, from 57.1% in patients <35 years to 51.6% in patients 35–37 years and to 35.8% in patients 38–42 years. A continued increase in CLBR from 15 oocytes retrieved was observed in older patients (≥38 years); from 41.3% at 15–19 oocytes retrieved to 53.4% at 20–24 oocytes retrieved to 58.7% at ≥25 oocytes retrieved. No equivalent benefit from retrieving more oocytes was observed in younger patients (<38 years), where the corresponding rates were 72.5%, 68.0%, and 78.8% in patients <35 years and 70.3%, 73.1%, and 71.5% in patients 35–37 years (Fig. 4A). The CLBR was similar for patients with AMH <15 pmol/l and ≥15 pmol/l (52.0% vs 50.9%), with a similar tendency towards a higher CLBR with higher number of oocytes retrieved in both subgroups (Fig. 4B). Subgroup analyses by dosing strategy (Supplementary Fig. S2) displayed similar patterns with some variations.

**Table 3.** Distribution of patients by age group, AMH group, and number of oocytes retrieved.

	Number of oocytes retrieved					Overall
	1–7	8–14	15–19	20–24	≥25	
**Age group (years)**						
<35, *n*	231	385	162	89	76	943
Percentage of age group <35 years, %	24.5	40.8	17.2	9.4	8.1	100.0
35–37, *n*	151	169	79	28	31	458
Percentage of age group 35-37 years, %	33.0	36.9	17.2	6.1	6.8	100.0
≥38, *n*	146	137	37	13	12	345
Percentage of age group ≥38 years, %	42.3	39.7	10.7	3.8	3.5	100.0
**AMH group (pmol/l)**						
<15, *n*	376	305	68	11	4	764
Percentage of AMH group <15 pmol/l, %	49.2	39.9	8.9	1.4	0.5	100.0
≥15, *n*	152	386	210	119	115	982
Percentage of AMH group ≥15 pmol/l, %	15.5	39.3	21.4	12.1	11.7	100.0

AMH = anti-Müllerian hormone.

### Cumulative live birth versus fresh cycle live birth

**Figure 5.**
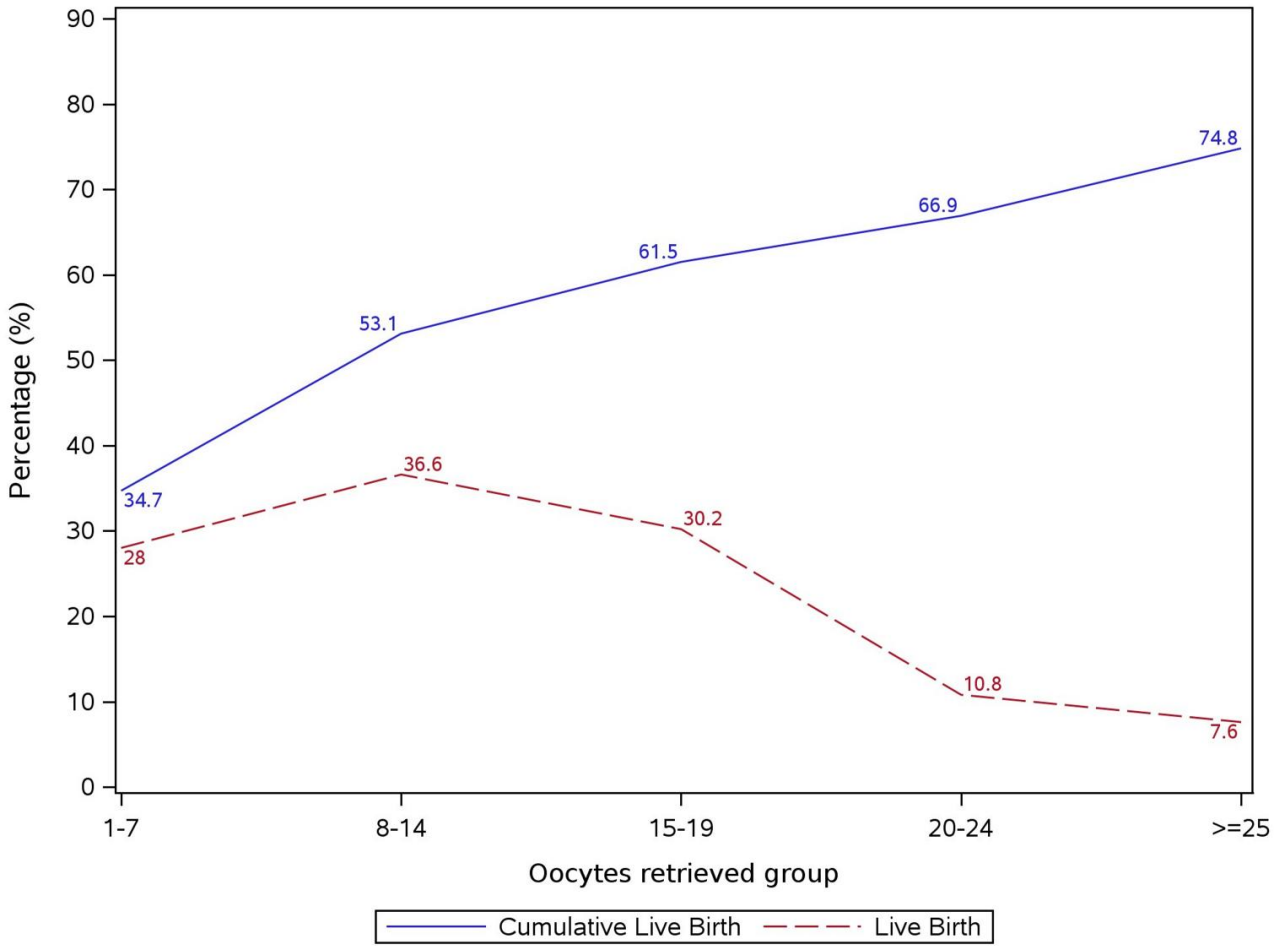
**Observed cumulative live birth rate versus fresh cycle live birth rate by number of oocytes retrieved (grouped).** The solid blue line represents observed cumulative live birth rates, and the dotted red line represents observed live birth rates.

A comparison of the observed fresh cycle LBR and the CLBR by number of oocytes retrieved demonstrated that the LBR decreased beyond 14 oocytes retrieved, while the CLBR continued to increase by number of oocytes retrieved (Fig. 5).

## Discussion

This pooled analysis of four randomized clinical trials evaluated the association between cumulative live birth and the number of oocytes retrieved in patients undergoing ovarian stimulation with follitropin delta. It demonstrated an increase in CLBR by the number of oocytes retrieved that started to plateau at 21–25 oocytes. Subgroup analyses by dosing strategy displayed similar trends with some variations. These variations may be attributable to differences in baseline characteristics of the trial populations, where patients treated with individualized follitropin delta on average were slightly younger and had lower BMI. Previous studies implied that CLBR continues to increase with the number of oocytes retrieved (Ji *et al.*, 2013; Drakopoulos *et al.*, 2016; Toftager *et al.*, 2017; Polyzos *et al.*, 2018; Law *et al.*, 2019; Neves *et al.*, 2023), although a plateau or decline beyond a certain number of oocytes retrieved, as observed for fresh cycle LBR, was also reported (Nelson *et al.*, 2016; Magnusson *et al.*, 2018). Some of the previous studies reporting a continuous increase in CLBR by number of oocytes retrieved used grouped categories of ovarian response with a highest cut-off at >15 oocytes retrieved (Ji *et al.*, 2013; Drakopoulos *et al.*, 2016; Toftager *et al.*, 2017). The same cut-off in this study would have led to the same conclusion. However, in the current study, the additional subgrouping of patients with responses at >15 oocytes and the analysis using fractional polynomials enabled further evaluation of CLBR at higher responses. Variations from the results of studies using similar approaches (Polyzos *et al.*, 2018; Law *et al.*, 2019) may be due to the lower number of patients in this study.

The difference between the optimum number of oocytes to achieve a live birth in the fresh cycle and the optimum number of oocytes to maximize the cumulative live birth was evident in the current study. The fresh cycle LBR declined substantially beyond 14 oocytes retrieved, while the CLBR continued to increase beyond 14 oocytes retrieved. This evidence is relevant when personalizing treatment for patients. Similar observations of diverging curves for LBR and CLBR after ∼15 oocytes were made in previous studies evaluating both outcomes (Ji *et al.*, 2013; Magnusson *et al.*, 2018, Polyzos *et al.*, 2018).

The CLBR decreased with increasing age, which is in agreement with previous studies (Polyzos *et al.*, 2018; Law *et al.*, 2019; Neves *et al.*, 2023). While Polyzos *et al.* observed a continuous increase in CLBR by the number of oocytes retrieved in all age groups, Neves *et al.* and Law *et al.*, noted a plateau around 25 oocytes for patients <35 years, whereas patients 35–44 years displayed a continued increase in CLBR beyond 25–30 oocytes. The current analysis suggests that patients <38 years do not benefit from oocyte numbers ≥20, since no evident increase in CLBR was observed, while an increase in CLBR from 20 oocytes was observed in patients ≥38 years. There was no difference in CLBR based on AMH level. Based on these findings, the optimum number of oocytes retrieved from a stimulation cycle is individual and dependent on patient characteristics. In younger patients with a higher risk of OHSS, who do not benefit from increased oocyte yields, the target number of oocytes could be lower than in older patients, who may increase their chances of achieving a live birth with a higher number of oocytes retrieved. However, it should be noted that in patients undergoing repeated frozen cycles, the success rates decreased as the number of repeated cycles increased.

As far as we are aware, this is the first pooled analysis based on patient-level data from randomized controlled trials to investigate the association between the number of oocytes retrieved and CLBR. It has the advantages of including a well-defined patient population, representing multiple centres and countries, and employing study procedures in accordance with current clinical practice.

From a clinical trial perspective, the inclusion of frozen cycles initiated up to 1 year after the start of stimulation is a satisfactory time period. On the other hand, the time limit prevents frozen blastocysts not used at the cut-off from inclusion in the analysis, which constitutes a limitation of the study. However, the majority of the patients (84.0%) in the study had either achieved a live birth or had no remaining blastocysts at the 1-year cut-off. Another limitation is the relatively small number of patients with more than 20 oocytes retrieved. In total, 249 patients (14.3%) had ≥20 oocytes retrieved, and 38 patients (2.2%) had ≥35 oocytes retrieved. In two of the included trials, no cycle cancellations were performed in case of excessive ovarian response; instead, triggering was performed with GnRH agonist, transfer was cancelled, and all blastocysts were cryopreserved. As a consequence, some patients had a very high number of oocytes retrieved. The low number of patients with a high ovarian response makes predictions unreliable in this range.

In conclusion, this analysis suggests an increase in CLBR with the number of oocytes retrieved up to a plateau starting at 21–25 oocytes retrieved following ovarian stimulation cycles with follitropin delta and subsequent frozen cycles. A benefit of an increase from 20 oocytes retrieved was age-dependent and evident in older but not in younger patients.

## Supplementary Material

deaf111_Supplementary_Figure_S1

deaf111_Supplementary_Figure_S2

## Data Availability

The data underlying this article will be shared on reasonable request to the corresponding author.

## References

[deaf111-B1] ClinicalTrials.gov [Internet]. *Identifier NCT03740737, Recombinant FSH Investigation in the Treatment of Infertility With Assisted Reproductive Technology (ART) (RITA-1)*. Bethesda, MD: National Library of Medicine (US). 29 Feb 2000, 14 November 2018. https://clinicaltrials.gov/study/NCT03740737 (8 March 2024, date last accessed).

[deaf111-B2] ClinicalTrials.gov [Internet]. *Identifier NCT03738618, Recombinant FSH Investigation in the Treatment of Infertility With Assisted Reproductive Technology (ART) (RITA-2)*. Bethesda, MD: National Library of Medicine (US), 29 Feb 2000, 24 October 2018. https://clinicaltrials.gov/study/NCT03738618 (8 March 2024, date last accessed).

[deaf111-B3] Devroey P , Polyzos NP, Blockeel C. An OHSS-Free Clinic by segmentation of IVF treatment. Hum Reprod 2011;26:2593–2597. 21828116 10.1093/humrep/der251

[deaf111-B4] Doody KJ , Scheiber MD, Grover SA, Foster ED, Elci OU, Malik P, Heiser PW. Ovarian stimulation with FE 999049 is efficacious and safe in women 35-42 years of age: primary findings of the RITA-2 registration trial [conference presentation abstract]. Fertil Steril 2023;120:e101.

[deaf111-B5] Drakopoulos P , Blockeel C, Stoop D, Camus M, de Vos M, Tournaye H, Polyzos NP. Conventional ovarian stimulation and single embryo transfer for IVF/ICSI. How many oocytes do we need to maximize cumulative live birth rates after utilization of all fresh and frozen embryos? Hum Reprod 2016;31:370–376. 26724797 10.1093/humrep/dev316

[deaf111-B6] Fernandez Sanchez M , Visnova H, Larsson P, Yding Andersen C, Filicori M, Blockeel C, Pinborg A, Khalaf Y, Mannaerts B; Rainbow Study Group. A randomized, controlled, first-in-patient trial of choriogonadotropin beta added to follitropin delta in women undergoing ovarian stimulation in a long GnRH agonist protocol. Hum Reprod 2022;37:1161–1174. 35451013 10.1093/humrep/deac061 PMC9156848

[deaf111-B7] Grover S , Foster ED, Heiser PW. Comparison of ovarian hyperstimulation syndrome (OHSS) rates resulting from conventional dosing versus personalized dosing of follitropin delta in ovarian stimulation, analysis of RITA, ESTHER-1 trials [conference presentation abstract]. Hum Reprod 2024;39 (Suppl. 1):i513.

[deaf111-B9] Ji J , Liu Y, Tong XH, Luo L, Ma J, Chen Z. The optimum number of oocytes in IVF treatment: an analysis of 2455 cycles in China. Hum Reprod 2013;28:2728–2734. 23887075 10.1093/humrep/det303

[deaf111-B10] Law YJ , Zhang N, Venetis CA, Chambers GM, Harris K. The number of oocytes associated with maximum cumulative live birth rates per aspiration depends on female age: a population study of 221 221 treatment cycles. Hum Reprod 2019;34:1778–1787. 31398253 10.1093/humrep/dez100

[deaf111-B11] Lobo R , Falahati A, Moley K, Pinborg A, Santos-Ribeiro S, Macklon NS, Jepsen IE. Oocyte yield and live birth rate after follitropin delta dosing and fresh embryo transfer: an individual patient data meta-analysis. Reprod Biomed Online 2025;50:104451. 39740370 10.1016/j.rbmo.2024.104451

[deaf111-B12] Magnusson A , Kallen K, Thurin-Kjellberg A, Bergh C. The number of oocytes retrieved during IVF: a balance between efficacy and safety. Hum Reprod 2018;33:58–64. 29136154 10.1093/humrep/dex334

[deaf111-B13] Nelson SM , Smith A, Tilling K, Lawlor DA. Optimal oocyte yield for cumulative live-birth rate: analysis of 257,398 IVF cycles and their linked fresh and frozen embryo transfers. Fertil Steril 2016;106:e191–e192.

[deaf111-B14] Neves AR , Montoya-Botero P, Sachs-Guedj N, Polyzos NP. Association between the number of oocytes and cumulative live birth rate: a systematic review. Best Pract Res Clin Obstet Gynaecol 2023;87:102307. 36707342 10.1016/j.bpobgyn.2022.102307

[deaf111-B15] Nyboe Andersen A , Nelson SM, Fauser BC, Garcia-Velasco JA, Klein BM, Arce J-C; ESTHER-1 Study Group. Individualized versus conventional ovarian stimulation for in vitro fertilization: a multicenter, randomized, controlled, assessor-blinded, phase 3 noninferiority trial. Fertil Steril 2017;107:387–396.e4. 27912901 10.1016/j.fertnstert.2016.10.033

[deaf111-B16] Olsson H , Sandström R, Grundemar L. Pharmacokinetic and pharmacodynamic properties of recombinant follicle-stimulating hormone (rFSH) derived from a human cell line compared with rFSH from a non-human cell line. J Clin Pharmacol 2014;54:1299–1307. 24800998 10.1002/jcph.328

[deaf111-B17] Olsson H , Sandström R, Bagger Y. Dose-exposure proportionality of a novel recombinant follicle-stimulating hormone (rFSH), FE 999049, derived from a human cell line, with comparison between Caucasian and Japanese women after subcutaneous administration. Clin Drug Investig 2015;35:247–253. 10.1007/s40261-015-0276-8 PMC4368841 25773354

[deaf111-B18] Polyzos NP , Drakopoulos P, Parra J, Pellicer A, Santos-Ribeiro S, Tournaye H, Bosch E, Garcia-Velasco J. Cumulative live birth rates according to the number of oocytes retrieved after the first ovarian stimulation for in vitro fertilization/intracytoplasmic sperm injection: a multicenter multinational analysis including approximately 15,000 women. Fertil Steril 2018;110:661–670.e1. 30196963 10.1016/j.fertnstert.2018.04.039

[deaf111-B20] Saket Z , Kallen K, Lundin K, Magnusson A, Bergh C. Cumulative live birth rate after IVF: trend over time and the impact of blastocyst culture and vitrification. Hum Reprod Open 2021;2021:hoab021. 34195386 10.1093/hropen/hoab021 PMC8240131

[deaf111-B21] Scheiber MD , Doody KJ, Grover SA, Foster ED, Elci OU, Malik P, Heiser PW. Ovarian stimulation with FE 999049 is efficacious and safe in women 18-34 years of age: primary findings of the RITA-1 registration trial [conference presentation abstract]. Fertil Steril 2023;120:e237.

[deaf111-B22] Shao F , Jiang Y, Ding S, Larsson P, Pinton P, Jonker DM. Pharmacokinetics and safety of follitropin delta in gonadotropin down-regulated healthy Chinese women. Clin Drug Investig 2023;43:37–44. 10.1007/s40261-022-01232-9 PMC9834375 36478528

[deaf111-B23] Shapiro BS , Daneshmand ST, Garner FC, Aguirre M, Ross R. Contrasting patterns in in vitro fertilization pregnancy rates among fresh autologous, fresh oocyte donor, and cryopreserved cycles with the use of day 5 or day 6 blastocysts may reflect differences in embryo-endometrium synchrony. Fertil Steril 2008;89:20–26. 17224151 10.1016/j.fertnstert.2006.08.092

[deaf111-B24] Steward RG , Lan L, Shah AA, Yeh JS, Price TM, Goldfarb JM, Muasher SJ. Oocyte number as a predictor for ovarian hyperstimulation syndrome and live birth: an analysis of 256,381 in vitro fertilization cycles. Fertil Steril 2014;101:967–973. 24462057 10.1016/j.fertnstert.2013.12.026

[deaf111-B25] Sunkara SK , Rittenberg V, Raine-Fenning N, Bhattacharya S, Zamora J, Coomarasamy A. Association between the number of eggs and live birth in IVF treatment: an analysis of 400 135 treatment cycles. Hum Reprod 2011;26:1768–1774. 21558332 10.1093/humrep/der106

[deaf111-B26] Toftager M , Bogstad J, Løssl K, Prætorius L, Zedeler A, Bryndorf T, Nilas L, Pinborg A. Cumulative live birth rates after one ART cycle including all subsequent frozen-thaw cycles in 1050 women: secondary outcome of an RCT comparing GnRH-antagonist and GnRH-agonist protocols. Hum Reprod 2017;32:556–567. 28130435 10.1093/humrep/dew358

[deaf111-B27] van der Gaast MH , Eijkemans MJ, van der Net JB, de Boer EJ, Burger CW, van Leeuwen FE, Fauser BC, Macklon NS. Optimum number of oocytes for a successful first IVF treatment cycle. Reprod Biomed Online 2006;13:476–480. 17007663 10.1016/s1472-6483(10)60633-5

