# Supplementary material for: One-year cumulative live birth rate associated with the number of oocytes in ovarian stimulation with follitropin delta: a pooled analysis of four randomized controlled trials
Source: Hum Reprod. 2025 Jun 12;40(8):1526–34. doi: 10.1093/humrep/deaf111 (PMC12314149; doi:10.1093/humrep/deaf111)
Supplement: deaf111_Supplementary_Figure_S1 [file deaf111_supplementary_figure_s1.pdf]

Human Reproduction, 2025, 1–1
https://doi.org/10.1093/humrep/deaf111
Supplementary Data

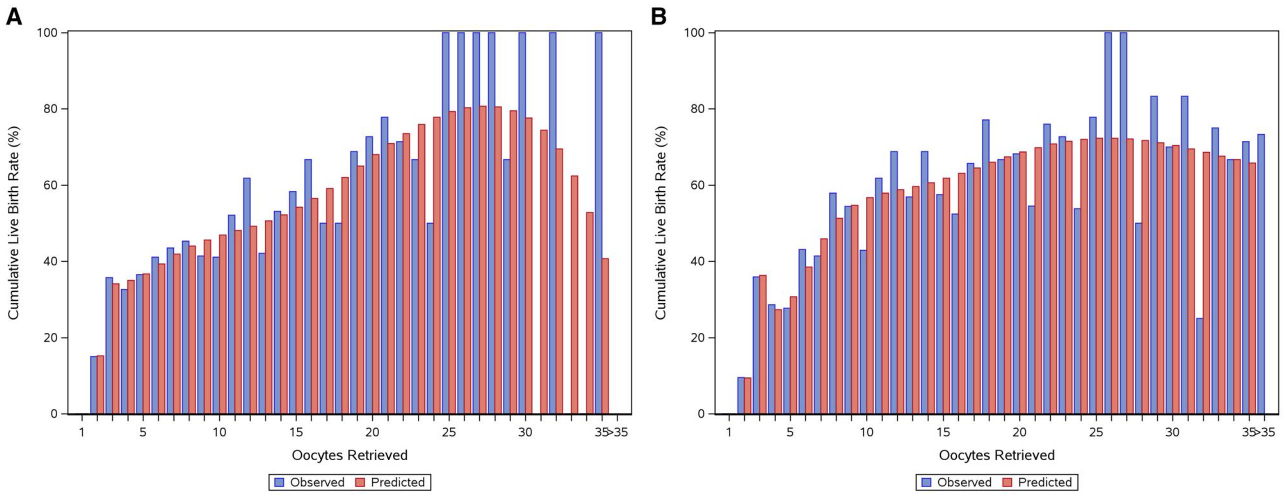

Supplementary Figure S1. Subgroup analyses of observed and predicted cumulative live birth rate by follitropin delta dosing strategy. (**A**) Patients with individualized fixed dosing of follitropin delta based on anti-Müllerian hormone and body weight (**B**). Patients with follitropin delta starting doses of 12 or 15 μg with potential dose adjustments during stimulation. Blue bars represent observed rates, and red bars represent predicted rates. Predicted cumulative live birth rates were obtained using a logistic regression analysis with fractional polynomials.
