# Supplementary material for: One-year cumulative live birth rate associated with the number of oocytes in ovarian stimulation with follitropin delta: a pooled analysis of four randomized controlled trials
Source: Hum Reprod. 2025 Jun 12;40(8):1526–34. doi: 10.1093/humrep/deaf111 (PMC12314149; doi:10.1093/humrep/deaf111)
Supplement: deaf111_Supplementary_Figure_S2 [file deaf111_supplementary_figure_s2.pdf]

Human Reproduction, 2025, 1–1
https://doi.org/10.1093/humrep/deaf111
Supplementary Data

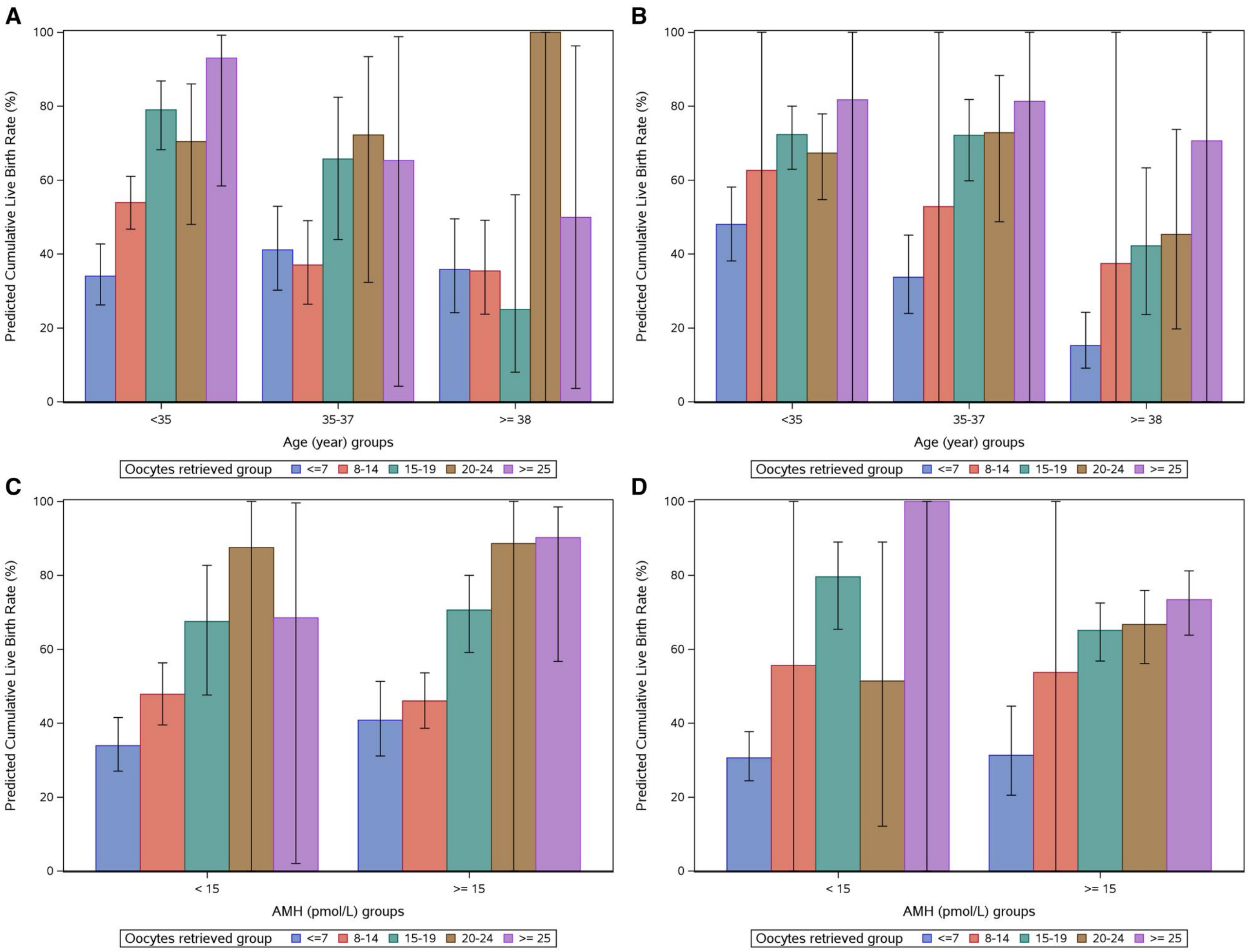

Supplementary Figure S2. Subgroup analyses of cumulative live birth rate by follitropin delta dosing strategy. (**A**) Cumulative live birth rate by number of oocytes retrieved (grouped) and age group—patients with individualized fixed dosing of follitropin delta based on anti-Müllerian hormone (AMH) and body weight (**B**) Cumulative live birth rate by number of oocytes retrieved (grouped) and age group—patients with follitropin delta starting doses of 12 or 15 µg with potential dose adjustments during stimulation (**C**). Cumulative live birth rate by number of oocytes retrieved (grouped) and AMH—patients with individualized fixed dosing of follitropin delta based on AMH and body weight (**D**). Cumulative live birth rate by number of oocytes retrieved (grouped) and AMH—patients with follitropin delta starting doses of 12 or 15 µg with potential dose adjustments during stimulation. Number of oocytes retrieved are grouped as ≤7 oocytes (blue bars), 8–14 oocytes (red bars), 15–19 oocytes (green bars), 20–24 oocytes (brown bars), and ≥25 oocytes (purple bars). Data are presented as percentage (95% CI). Predicted cumulative live birth rates were obtained using logistic regression analyses.

© The Author(s) 2025. Published by Oxford University Press on behalf of European Society of Human Reproduction and Embryology.
This is an Open Access article distributed under the terms of the Creative Commons Attribution License (https://creativecommons.org/licenses/by/4.0/), which permits unrestricted reuse, distribution, and reproduction in any medium, provided the original work is properly cited.
Human Reproduction, 2025, 00, 1–9
https://doi.org/10.1093/humrep/deaf111
Original Article
